# Differences in Aggression and Alcohol Use among Youth with Varying Levels of Victimization and Popularity Status

**DOI:** 10.1007/s10964-022-01649-7

**Published:** 2022-07-01

**Authors:** Sarah T. Malamut, Molly Dawes, Tessa A. M. Lansu, Yvonne van den Berg, Antonius H. N. Cillessen

**Affiliations:** 1. Behavioural Science Institute, Radboud University, Nijmegen, Netherlands; 2. INVEST Research Flagship Center/Psychology, University of Turku, Turku, Finland; 3. College of Education, University of South Carolina, Columbia, SC, USA

**Keywords:** Victimization, Popularity, Aggression, Alcohol use, Adolescents, Latent profile analysis

## Abstract

Awareness that high-status adolescents can be targets of aggression has grown in recent years. However, questions remain about the associations of the confluence of victimization and popularity with adjustment. The current study fills this gap by examining the joint and unique effects of victimization and popularity on aggression and alcohol use. Participants were 804 Dutch adolescents (50.2% boys, *M*_age_ = 13.65) who were followed for one year. High-status victims were more aggressive and drank more alcohol than lower-status victims. High-status victims were also more proactively and indirectly aggressive and self-reported more bullying than high-status non-victims. Thus, the findings demonstrated a conjoined risk of victimization and popularity for some types of aggression.

## Introduction

Victimization is a serious problem affecting youth and has been identified as a public health crisis in several countries (Gladden et al., 2014). Efforts to understand the consequences of victimization are complicated by the fact that victimized youth are not a homogenous group (e.g., Scholte et al., 2013). For example, a small but growing body of literature has found heterogeneity in social status among victimized youth, contrary to common stereotypes that victims are disempowered youth (e.g., see Dawes & Malamut, 2020, for a review). A recent paper (Malamut et al., 2021) identified six groups of victims and non-victims differing in peer status, including a group of high-status victims that experienced internalizing symptoms. The goal of the current study was to further understand this complex group of high-status (i.e., popular) victims by examining their concurrent and prospective behaviors, given the dearth of research on how the confluence of victimization and popularity impacts adjustment (Dawes & Malamut, 2020). To create clear comparisons, the current study focused on victims and non-victims who were either low or high in popularity, resulting in four groups: high-status victims, lower-status victims, high-status non-victims, and lower-status non-victims. The current study focused on aggression and alcohol use, which are both positively associated with popularity (Mayeux et al., 2008; Salmivalli et al., 2021), but are also two common responses to peer victimization (for aggression, see, e.g., Malamut & Salmivalli, 2021; for alcohol use, see, e.g., Manglio et al., 2017). Given that both victimization and popularity can be risk factors for these behaviors, the current study directly examined whether they jointly conferred risk for aggression and alcohol use compared to popularity or victimization alone.

## Differences in Aggression Across Victim Types

Aggression can be motivated in different ways. A common distinction is between proactive aggression that serves clearly defined goals (e.g., gaining resources, establishment of dominance) and reactive aggression which is impulsive, retaliatory, and intense (e.g., Connor et al., 2019). Aggression can also differ in form. Here a distinction is between direct aggression (e.g., inflicting physical harm or verbally intimidating a victim directly) and indirect aggression (e.g., strategically hurting someone’s relationships and social position by weakening their social ties through gossip or exclusion). In this study, both the functions (proactive vs. reactive) and forms (indirect vs. direct) of aggression were examined. In addition, bullying behavior was also examined, which is considered a goal-directed, more severe subtype of aggression (Volk et al., 2014). Victimized youth can also engage in bullying themselves (“bully-victims”; Yang & Salmivalli, 2013) or become more likely to bully over time (Malamut & Salmivalli, 2021).

Both popular and victimized youth behave aggressively. For instance, popular youth may use aggression to demonstrate power and social dominance to classmates, or to proactively ward off potential social competitors (e.g., Pellegrini et al., 2011). Indeed, recent studies have shown that aggression is a tool that youth use to maintain or gain status (e.g., van den Berg et al., 2019). Victimized youth may be aggressive in order to retaliate or to attempt to save themselves from continued or future aggression. This explains why victimization is also positively associated with aggression (e.g., Yeung & Leadbeater, 2007).

Although both popularity and victimization independently predict aggression, few studies have examined how they jointly relate to aggression, and the different ways in which the aggression is enacted. High-status and lower-status victims may use aggression differently, stemming from different motivations as well as different skills and/or resources for distinct forms of aggression. It has been proposed that high-status youth have “more to lose” than low-status youth when they are victimized, given their high status and the investments they made for it (Faris & Felmlee, 2014). Indeed, high-status victims had worse adjustment scores than lower-status victims, such as significantly larger increases in anger (Faris & Felmlee, 2014). High-status victims may feel that their social standing has been threatened and therefore use aggression in strategic ways to reestablish their social dominance and protect their social position. In addition, their central position in the peer group may enable them to use the peer group to their advantage in harming others (i.e., indirect aggression; Hawley, 2003). Thus, high-status victims may be more likely to use proactive, goal-directed aggressive behaviors that are related to gaining, maintaining, or demonstrating their status (i.e., bullying, proactive aggression, indirect aggression) than lower-status victims. Preliminary support for this expectation comes from a recent study demonstrating that youth with high levels of victimization *and* popularity had elevated levels of relational aggression (i.e., rumor spreading, excluding others) one year later (Malamut et al., 2020a).

In contrast to goal-directed proactive aggression, reactive aggression is impulsive, “hot-headed” aggression in response to a perceived threat. It can indicate deficiencies in emotion regulation and social competence (e.g., Hubbard et al., 2010) and is negatively correlated with popularity (Stoltz et al., 2016; van den Berg et al., 2019). Although some high-status youth may be reactively aggressive (Stoltz et al., 2016), low-status youth seem to exhibit the highest levels of reactive aggression and increased reactive aggression is related to even lower status (Stoltz et al., 2016; van den Berg et al., 2019). Lower-status victims may not have the social resources to engage in goal-directed proactive aggression, but may instead react aggressively to perceived threats. Indeed, youth who are perpetrators and targets of bullying (“bully-victims”; Yang & Salmivalli, 2013), generally have low-status, poor social skills, and are rejected by peers (e.g., Guy et al., 2017, 2019). Thus, whereas high-status victims may have the social skills and resources to be aggressive strategically, lower-status victims are more likely to “lash out” impulsively in response to provocations. Therefore, lower-status victims were expected to be more reactively aggressive than high-status victims.

Whereas there are clear expectations for how the aggressive behaviors discussed above would relate to victimization and popularity, it is not as clear for direct aggression. Direct aggression, whether physical or verbal (e.g., hitting, name calling), can be goal-directed and instrumental or impulsive and reactionary (e.g., Prinstein & Cillessen, 2003). High levels of popularity have been associated with direct aggression concurrently (e.g., Waasdorp et al., 2013) and over time (e.g., Malamut et al., 2020b), which suggests that it can be used by high-status youth in instrumental ways. Thus, high-status victims may use direct aggression to publicly reaffirm their dominance in the peer group if they feel their position is threatened. Alternatively, lower-status victims may react to provocations with direct forms of aggression (e.g., pushing or yelling; Prinstein & Cillessen, 2003). Therefore, a priori hypotheses were not made regarding whether high-status victims would use direct aggression more or less than lower-status victims.

To better understand how high status and victimization may jointly impact aggression, it is also important to compare high-status victims to high-status non-victims. As indicated, popularity in general is a risk factor for aggressive behavior as a means to maintain or demonstrate status (van den Berg et al., 2019). Insofar as victimization can lead to aggression to defend one’s status, youth who are *both* popular and victimized may be at heightened risk for aggression compared to high-status non-victims. That is, high-status victims may engage in even more aggressive behavior, as they feel like their social position is threatened (compared to high-status non-victims, who are not experiencing a threat to their status).

## Differences in Alcohol Use Across Victim Types

As with aggression, alcohol use is a behavior that has been found to be positively associated with both popularity (e.g., Guyll et al., 2014; Tucker et al., 2013) and victimization (Maniglio, 2017). Specifically, drinking alcohol can be considered a “popularity-enhancing” behavior in early adolescence, meaning that drinking can signal to peers that they are cool (Gommans et al., 2017). During this developmental period, the opinions of the peer group become more important to youth, as youth become more independent from adults (e.g., Laursen & Veenstra, 2021). High-status early adolescents display and increase behaviors that are seen as deviant by adults (Moffitt, 1993), including alcohol use (Allen et al., 2005). That is, popular youth may drink more alcohol as it suggests to others that they are mature, autonomous, and willing to rebel (e.g., maturity gap hypothesis; Moffitt, 1993). In addition, popular youth likely have more opportunities to drink alcohol (e.g., invitations to parties; Schwartz & Gorman, 2011).

Peer victimization is also a risk factor for alcohol use, but for different reasons. Victimization may be associated with substance use as a maladaptive mechanism to cope with the painful experience (i.e., stress-coping model; Wills & Filer, 1996). Indeed, studies have found associations between victimization and alcohol use over time, mediated by internalizing problems (Earnshaw et al., 2017; Rowe et al., 2019). In a recent review, Maniglio (2017) reported inconsistent associations between victimization in general (not just by peers) and alcohol use in the literature. Studies found positive, negative, and nonsignificant associations. However, there was a more consistent positive association with alcohol use for bullying victimization than for other types (e.g., violent victimization). Taking both the popularity and victimization elements together, high-status victims may drink more alcohol (both concurrently and longitudinally) than lower-status victims given their two possible motives (reaffirming their high status and/or coping with the victimization) and more opportunities to do so (e.g., access to parties).

## Current Study

There is a dearth of research examining the adjustment of high-status victims. The goal of this study was to examine how victimization and popularity jointly predicted concurrent and prospective aggression and alcohol use. This study built on and extended previous work (Malamut et al., 2021), by examining these externalizing behaviors in four groups of youth: high-status victims, lower-status victims, high-status non-victims, and lower-status non-victims. High-status victims were expected to use more behaviors intended to maintain their status (i.e., bullying, proactive aggression, indirect aggression, and alcohol use) than lower-status victims. In contrast, lower-status victims were expected to have higher levels of reactive aggression than high-status victims. Given that both high-status and lower-status victims may use direct aggression for different reasons, no hypothesis was generated for this comparison. Lower-status victims were expected to use more reactive aggression and direct aggression than either non-victim group, given that these behaviors could be in response to their victimization experiences. Both high-status victims and high-status non-victims were expected to engage in higher levels of aggression and alcohol use than lower-status victims and lower-status non-victims. High-status victims were expected to engage in more aggression and alcohol use than high-status non-victims, because they may feel the need to protect their status from perceived threats.

## Method

### Participants and Procedure

Participants were recruited as part of the Kandinsky Longitudinal Study (KLS), which began in 2010 at the request of the school to monitor the social well-being of students (van den Berg et al., 2019). This study includes data from participants during waves 6 and 7 (i.e., years 2015 and 2016). Each year, the head of the school formally requested the data collection and claimed responsibility for the parental consent procedure. In the beginning of the school year, the head of the school gave parents a detailed letter outlining the goal and procedures of data collection, and informed parents that they could exclude their child from participation. No parents objected to the participation of their child. Adolescents were informed of the details of the study and were asked for active assent at each assessment. No students declined to participate at any stage of the assessment. Anonymized data was made available to the researchers for scientific purposes. This procedure was approved by the Institutional Review Board of the Behavourial Science Institute at Radboud University (Protocol Number: ECG2012–2505-038; Project Title: “Sociometry as a method to measure social relationships among children and adolescents”). Participants were 833 Dutch adolescents in grades 7–9. Twenty-nine students were absent on the day of data collection at T1, resulting in a sample of 804 adolescents (50.2% boys, *M*_age_ = 13.65, 90.2% born in the NL).

### Measures

Participants completed computerized peer nominations and self-report questionnaires. For each peer nomination item, participants could nominate an unlimited number of classmates, but not themselves. Classmates’ names were shown in random order between participants, but in the same order across peer nomination questions within participants. Nominations received were counted for each student and standardized within classrooms.

#### Victim subtypes measures

##### Victimization

Self-reported victimization was assessed using an extended version of the Olweus’ Bully-Victim questionnaire (Solberg & Olweus, 2003). The questionnaire included six items about experiences of victimization (e.g., “how often have other students ignored you”), which were rated on a scale from 1 (*never*) to 5 (*several times a week)*. The items were averaged to form one victimization score (Cronbach’s α = 0.71). Peer-reported victimization was assessed with the item “who in your class are bullied by others?”

##### Popularity

Popularity was assessed with two peer nomination items, namely “who in your class are most popular” and “who in your class are least popular.” These items were used as separate variables in the latent profile analysis (Marks et al., 2022).

#### Outcome measures

##### Bullying

Self-reported bullying was assessed using an extended version of the Olweus’ Bully-Victim questionnaire (Solberg & Olweus, 2003). The questionnaire included six items about experiences of bully perpetration (e.g., “how often have you pushed, kicked, or beaten other students?”), which were rated on a scale from 1 (*never*) to 5 (*several times a week*) and averaged to form one bullying score (Cronbach’s α = 0.63 and 0.70 at T1 and T2, respectively). Peer-reported bullying was assessed with the item: “who in your class bully others”.

##### Proactive and reactive aggression

Proactive aggression was assessed with the nomination item: “who try to reach their goals by using aggressive behavior. These classmates intimidate, manipulate, or bully others to get admiration, respect or objects” (Stoltz et al., 2016). Reactive aggressive youth were nominated by classmates for the item: “who feel threatened or attacked easily (even though this might not have been intended). These classmates are not able to control their behavior and feelings and react with aggressive behavior, like yelling or hitting” (Stoltz et al., 2016).

##### Indirect and direct aggression

Indirect aggression was assessed with two nomination items that were standardized within classroom and averaged together: “who in your class say nasty things or gossip about others?” and “who in your class exclude others or ignore others?”. Direct aggression was also assessed with two nomination items that were standardized within classroom and averaged together: “who in your class calls others names?” and “who in your class pushes, kicks, or hits others?”

##### Alcohol use

Participants self-reported their alcohol use with the item: “in the last 30 days, on how many days did you drink alcohol?” (e.g., Gommans et al., 2016). Responses ranged from 0 (*never*) to 6 (*all 30 days*).

### Analytic Plan

Victim types were identified with latent profile analysis (LPA) with tidyLPA in R (Rosenberg et al., 2018) in a previous study (Malamut et al., 2021). Details of the identification process are publicly available (https://doi.org/10.17026/dans-zj8-kba2) and briefly summarized herein. We used both peer- and self-reports to identify victimized youth in the analysis. Peer-reported victimization reflects youth’s reputation as a victim in the peer group. Self-reported victimization represents youth’s own experiences of being targeted. Because the concordance between the two perspectives is moderate (e.g., Dawes et al., 2017), using only one perspective might misclassify some victims as non-victims.

An initial latent profile analysis with four variables (peer- and self-reported victimization, peer nominations for most and least popular) identified self-identified victim groups that included some youth who actually did not self-report any victimization. To address this problem, a binary variable was created for self-reported victimization. Participants were classified as self-reported victims if they reported victimization across all forms for a frequency of at least 2 to 3 times a month, which is the recommended threshold to identify self-reported victims of bullying (Solberg & Olweus, 2003). By this criterion, 280 participants self-reported being victimized and 524 participants did not.

A latent profile analysis was then conducted for the victimized group and non-victimized group separately, using three variables: peer-nominated victimization, peer nominations for most popular, and peer nominations for least popular. The number of classes for each LPA was based on fit indices, class size, and the interpretability of the classes (Nylund et al., 2007; Nylund-Gibson & Choi, 2018). Using this procedure, the previous study identified six victim subtypes (see Malamut et al., 2021), which were reclassified to directly test the hypotheses in the current study. Youth were considered highly victimized if they had high levels of self-reported victimization and/or high levels of peer-reported victimization. This decision was made to avoid misclassifying some victims as non-victims, as both self- and peer-reported victimization have important implications for youth’s adjustment (Scholte et al., 2013). The resulting four profiles were: (1) high-status victims (*n* = 63, 7.8%), (2) lower-status victims^1^ (*n* = 282, 35.1%), (3) high status non-victims (*n* = 78, 9.7%), and (4) lower status non-victims (*n* = 381, 47.4%) (see Table 1).

Associations with aggression and alcohol use were determined with multilevel mixed-effect linear regression analyses with maximum likelihood estimation. At both time points (T1 and T2) and for each dependent variable, an unconditional model was first conducted to calculate the intraclass correlation coefficients (ICCs) indicating the variance partitioned within and between classrooms. The ICCs for aggression were 0.009 (self-reported bullying) or less, meaning that less than 1% of the variance in aggression was between classrooms. This was expected given that all peer-nominated variables were standardized within class-room. For alcohol use, the ICC was 0.132 at T1 and 0.154 at T2, meaning that 13.2 to 15.4% of variance in alcohol use was between classrooms, suggesting that multilevel analyses were warranted. Thus, multilevel analyses were conducted, with students at Level 1 and classrooms at Level 2.

In order to test the hypothesized group differences, a series of models were conducted in which the reference group changed. To account for multiple group comparisons, *p*-values were adjusted with the Holm-Bonferroni correction (Abdi, 2010; Holm, 1979). Given significant gender differences in victim group membership (see Malamut et al., 2021), gender was controlled for (0 = boys, 1 = girls) in the analyses. The adjusted means after model estimation are reported in Table 3.

## Results

### Descriptive Statistics

Table 2 reports the means, standard deviations, and correlations between study variables at T1 and T2. Given the large sample size, correlations are considered significant at *p* < 0.01. Self- and peer-reported victimization were moderately and positively correlated. Self-reported victimization was related to being named as least popular, but not to most popular nominations. Self-reported victimization was positively related to proactive aggression and direct aggression at T1, and to reactive aggression and self-reported bullying at T1 and T2. There was no significant association between self-reported victimization and alcohol use at either time.

Peer-nominated victimization was positively related to least popular nominations and negatively related to most popular nominations. At both time points, peer-nominated victimization was positively related to reactive aggression and negatively to indirect aggression.

### Differences in Aggressive Behavior and Alcohol Use

#### Self-reported bullying

High-status victims self-reported bullying others significantly more than lower-status victims at T1 (*p* < 0.001), but not at T2 (*p* = 0.018; Holm–Bonferroni adjusted threshold for significance at *p* = 0.0167; see Table 3). High-status victims also reported more bullying than non-victims at both time points, irrespective of the status of the non-victims (*p*s < 0.001). Lower-status victims reported significantly more bullying than lower-status non-victims at both time points (*p*s < 0.001), and compared to higher-status non-victims at T1 (*p* = 0.004). The non-victim groups did not differ significantly at either time point (*ps* > 0.083). In sum, victims – both high-status and lower-status – self-reported more bullying behavior than non-victims groups, with high-status victims reporting the highest level of bullying.

#### Peer-reported bullying

High-status victims and high-status non-victims did not differ in levels of peer-nominated bullying (*p*s > 0.032; adjusted threshold for significance at *p* = 0.025). Similarly, lower-status victims and lower-status non-victims did not differ in their levels of peer-nominated bullying at either time point (*p*s > 0.222). However, regardless of their victimization, high-status youth scored significantly higher on peer-reported bullying than lower-status youth (*ps* < 0.001). In other words, the two high-status groups were more likely to be seen as bullies by peers than the two lower-status groups.

#### Proactive aggression

At T1, all four groups significant differed from each other in proactive aggression. High-status victims were seen as most proactively aggressive, followed by high-status non-victims, lower-status victims, and lower-status non-victims (*ps* < 0.045). At T2, high-status youth scored significantly higher on proactive aggression than lower-status youth, irrespective of youth’s victimization (*p*s< 0.001). In sum, high-status victims engaged in the highest levels of proactive aggression at T1 of all groups, including high-status non-victims. Over time, high-status victims no longer differed from high-status non-victims in proactive aggression.

#### Reactive aggression

No significant difference was found between lower-status victims and high-status victims in peer-nominated reactive aggression at T1 (*p* = 0.018; adjusted threshold for significance at *p* = 0.0125). At T2, however, lower-status victims scored significantly higher in reactive aggression than high-status victims (*p* = 0.005). At both T1 and T2, lower-status victims had significantly higher levels of reactive aggression than both non-victim groups (*p*s< 0.001). No significant differences were found in reactive aggression between high-status non-victims and lower-status non-victims at either time, *p*s > 0.457. Thus, lower-status victims were more reactively aggressive than most of their peers.

#### Indirect aggression

High-status victims engaged in more indirect aggression than lower-status victims at both time points (*p*s < 0.001). They also scored higher on indirect aggression than high-status non-victims at T1 (*p* = 0.01), but no longer at T2. High-status non-victims had the second highest levels of indirect aggression, significantly higher than lower-status victims and lower-status non-victims at both time points (*p*s < 0.001). Lower-status victim and lower-status non-victims did not differ significantly from one another at either time point (*p*s > 0.122). Overall, the high-status groups were more likely to be nominated as indirectly aggressive than the lower-status groups. Further, high-status victims had even higher levels of indirect aggression than high-status non-victims at T1.

#### Direct aggression

High-status victims and high-status non-victims did not differ in their levels of direct aggression at either time point (*p*s > 0.028; adjusted *p*-value for comparison = 0.025). Similarly, lower-status victims and lower-status non-victims did not differ in their direct aggression at T1 or T2 (*ps* > 0.163). Regardless of their victimization, high-status youth scored significantly higher on direct aggression compared to lower-status youth (*ps* < 0.001). In other words, the two high-status groups were more likely to be seen as directly aggressive compared to the two lower-status groups.

#### Alcohol use

High-status victims and high-status non-victims reported using alcohol significantly more than the lower-status victim and non-victim groups at both time points (*p*s < 0.001). However, the two high-status groups did not differ from one another at either time point (*ps* > 0.162). Similarly, the two lower-status groups (victims and non-victims) did not significantly differ at T1 nor T2 (ps > 0.51). Thus, high-status youth – whether victimized or not – were more likely to use alcohol compared to their lower-status peers.

## Discussion

Aggression and alcohol use can represent significant detrimental barriers to optimal functioning for adolescents (e.g., Sullivan et al., 2006). The current study focused on two risk factors: victimization and popularity. Using a person-oriented approach, concurrent and prospective differences in aggression and alcohol use were examined between subgroups of victims who varied in popularity. High-status victims were more aggressive (for all forms of aggression except reactive aggression) and drank more alcohol than lower-status victims. Although high-status victims and high-status non-victims did not differ in alcohol use, there was some support that high-status victims were even more aggressive than high-status non-victims.

### Differences in Aggression Across Victim Types

The first goal of the current study was to examine how popularity and victimization may jointly confer risk for aggressive behavior. It is well established that some victimized youth also perpetrate aggression (e.g., “bully-victims”: Yang & Salmivalli, 2013). These youth are typically rejected by peers and reactively aggressive (Salmivalli & Nieminen, 2002). Evidence for this type of bully-victim was found in the current study, as lower-status victims had higher levels of reactive aggression than other youth - this finding may be particularly driven by youth who both report being victimized *and* are seen by peers as victimized (i.e., convergent victims; see Supplemental Table 1). However, a key finding was the existence of a different type of bully-victim: one with high popularity. High-status victims had higher concurrent and prospective levels of aggression than lower-status victims on peer-reported bullying as well as proactive, indirect, and direct aggression. These results indicate variability in the psychosocial profiles of bully-victims (see also Kennedy, 2021). The heterogeneity of bullies and victims is recognized in the literature (e.g., Malamut et al., 2020c; Scholte et al., 2013) and the current investigation indicates that such recognition should also apply to bully-victims. The results also emphasize the importance of considering multiple informants, as high-status victims would not have been identified as bully-victims using only peer-reports of victimization.

Another key finding was that high-status victims engaged in more aggressive behavior (depending on form and function) than high-status non-victims. Specifically, high-status victims self-reported more concurrent and prospective bullying and were seen by peers as more proactively and indirectly aggressive concurrently. The positive association between status and aggressive behavior is well-established (e.g., Salmivalli et al., 2021). The current study examined whether the victimization experience of those youth at the top of the social hierarchy would amplify this association. The findings support the premise that high-status victims may be strategically aggressive (i.e., proactive, indirect) to recoup perceived losses in social power or popularity due to their victimization (see Dawes & Malamut, 2020, for discussion).

These findings have implications for intervention. For example, interventions have been less successful at reducing bullying by popular youth than by youth with average or low popularity (Garandeau et al., 2014). This could be due to popular youth’s own victimization experiences. That is, it may be difficult to convince high-status victims to change their aggressive behavior when they have “a lot to lose”. Because their victimization threatens their status, they may feel justified to retaliate through proactive or indirect aggression. These youth may not refrain from bullying because they see it as a viable means to reclaim or assert their position at the top of the social ladder. When considering why interventions may not be effective for popular bullies, it is critical to ascertain whether they also report being victimized and whether reducing their victimization experiences might help to reduce their aggression.

Additional attention should be given to the importance of using multiple informants to understand behavioral risks. High-status victims self-reported higher levels of bullying than high-status non-victims but there was no difference in their bullying according to peers. Whether or not youth admit to bullying also has important ramifications for intervention. For example, targeted interventions have made distinctions between confronting and non-confronting approaches when intervening with a perpetrator (e.g., Johander et al., 2022), but an important first step is whether a perpetrator even acknowledges their bullying behavior. In addition, lower-status victims self-reported more concurrent bullying than high-status non-victims, despite not being seen as a bully by their peers. This is consistent with previous research that found some youth who self-identified as a bully but did not have that reputation (e.g., Obermann, 2011). This underscores the importance of considering both self- and peer-reports of bullying and victimization. In addition, more research is needed to understand the unique group of youth who say they bully others but are not seen as bullies by their peers.

### Differences in Alcohol Use Across Victim Types

Victimization and popularity are both risk factors for alcohol use (Manglio, 2017; Mayeux et al., 2008). Therefore, the second goal of this study was to compare the four profiles on alcohol consumption. As expected, in addition to being more aggressive, high-status victims were also more likely than lower-status victims to drink alcohol, both concurrently and later. Unexpectedly, high-status victims were not more likely to drink alcohol than high-status non-victims. Thus, the results indicated that high-status youth, regardless of their victimization status, were more likely to use alcohol than lower-status peers. These findings are consistent with past research demonstrating that popularity confers risk for alcohol use (e.g., Mayeux et al., 2008), as popular youth may have easier access to alcohol. However, the findings did not support that victimization exacerbated the risk for frequent alcohol use.

While differences were not found in the rate of alcohol consumption, it is still critical to consider the joint risk of popularity and victimization on alcohol use, for two reasons. First, the association between victimization and alcohol use may be more complicated (i.e., indirect effects via internalizing problems; Manglio, 2017). Second, distinctions can be made between so-called “normative” experimentation with alcohol use in adolescence (Petit et al., 2013) and more severe, risky alcohol use, which was not accounted for in the current study. Given the significant public health concerns associated with alcohol in adolescence (e.g., Peacock et al., 2018), it is critical to understand intra- and interpersonal factors that lead to severe or risky alcohol use at this age. Prior research has demonstrated that youth’s drinking motives play an important role in alcohol-related problems (Topper et al., 2011). To facilitate timely prevention or intervention, it is essential to know whether high-status victims and high-status non-victims have different drinking motives. For example, there could be distinctions between “proactive drinking” (i.e., drinking to gain status) compared to “reactive drinking” (i.e., drinking to cope with pain).

### Strengths, Limitations, and Future Directions

This study advanced our understanding of how victimization and popularity are jointly and uniquely related to concurrent and prospective aggression and alcohol use. Strengths of this study are a longitudinal design and an advanced classification method. The current study included multiple informants and examined several forms of aggression. Distinct and unique findings emerged across groups that may have been obfuscated if only one type of aggression had been examined.

This study also had some limitations. First, the design did not allow us to detect immediate reactions to feeling victimized (e.g., aggression, alcohol use). Different methodologies (e.g., daily diaries) are needed to see how these processes unfold in real time. Second, assumptions were made that aggression and alcohol use are reactions to experiences of victimization, but the current study could not test the underlying mechanisms behind these associations. Longitudinal mediational analyses are needed to further disentangle the complex associations of victimization with aggression and alcohol use over time. For example, future research could examine the role of critical social cognitions (e.g., status motivations; attributions for victimization; rumination) in the initiation and sustainment of aggression and alcohol use.

Third, it was not tested whether the behaviors of high-status victims (aggression, alcohol use) actually helped them stay popular or reduced their victimization. Do high-status victims who engage in aggression or substance use indeed maintain their status? Future research also could address whether the aggressive strategies of high-status victims (alone or in combination with prosocial strategies: Hawley, 2003) help them prevent decreases in popularity when they are victimized, or reduce their victimization experiences.

Fourth, some group differences may not have been detected due to limited power (some groups were small). Future studies with larger group sizes may detect additional differences between high-status victims and high-status non-victims. Future research could also examine whether other characteristics (e.g., social goals) differentiate these two groups, which would further benefit intervention. Fifth, the measure of alcohol use was limited to one item; future research is needed with stronger measurement (see Finan et al., 2020). Sixth and finally, the sample was rather homogenous. Future research should examine whether our findings generalize to other contexts and more diverse samples.

## Conclusion

Not all victimized youth are equally at risk for adjustment difficulties. The current study elucidated the potential role of social status by comparing youth with different combinations of high and low levels of victimization and popularity on concurrent and prospective aggression and alcohol use. The findings confirm that “bully-victims” are not a homogenous group. A group of lower-status victims were identified who were high in reactive aggression (typical for bully-victims), but also a group of high-status victims were identified who were high in all other measures of aggression. For alcohol use, popularity remained a stronger predictor than victimization. The findings suggest that the victimization experiences of high-status youth warrant attention as their experiences may contribute to a cycle of aggression in the peer group which has implications for the adjustment of all youth.

## Supplementary Material

Table S1

## Figures and Tables

**Table 1:** Comparison of independent variables by victim subgroups

	Victims		Non-victims	
	High-status Victims (*n* = 63)	Lower-status Victims (*n* = 282)	High-status Non-victims (*n* = 78)	Lower-status Non-victims (*n* = 381)
Victimization				
Self-report	1.86 (0.42)	1.73 (0.55)	1.12 (0.13)	1.14 (0.13)
Peer-report	−0.37 (0.16)	0.48 (1.44)	−0.36 (0.12)	−0.22 (0.51)
Most popular	1.88 (0.65)	−0.48 (0.37)	1.88 (0.73)	−0.33 (0.44)
Least popular	−0.58 (0.13)	0.67 (1.33)	−0.63 (0.14)	−0.28 (0.43)

**Table 2:** Means, standard deviations, and correlations for main study variables

	1.	2.	3.	4.	5.	6.	7.	8.	9.	10.	11.	12.	13.	14.	15.	16.	17.	18.
Time 1																		
1. Self-reported victimization	–																	
2. Peer-reported victimization	0.27^***^	–																
3. Most popular	0.01	−0.23^***^	–															
4. Least popular	0.13^***^	0.61^***^	−0.41^***^	–														
5. Self-reported bullying	0.44^***^	−0.02	0.15^***^	−0.13^***^	–													
6. Peer-reported bullying	0.06	−0.08	0.44^***^	−0.22^***^	0.23^***^	–												
7. Proactive aggression	0.10^**^	0.03	0.32^***^	−0.08	0.23^***^	0.63^***^	–											
8. Reactive aggression	0.29^***^	0.62^***^	−0.10^**^	0.39^***^	0.06	0.14^***^	0.27^***^	–										
9. Indirect aggression	0.09	−0.11^**^	0.49^***^	−0.25^***^	0.20^***^	0.68^***^	0.47^***^	0.12^***^	–									
10. Direct aggression	0.09^**^	−0.02	0.47^***^	−0.21^***^	0.26^***^	0.70^***^	0.80^***^	0.20^***^	0.47^***^	–								
11. Alcohol use	0.02	−0.08	0.23^***^	−0.15^***^	0.22^***^	0.13^***^	0.16^***^	−0.06	0.14^***^	0.19^***^	–							
Time 2																		
12. Self-reported bullying	0.28^***^	−0.04	0.09	−0.09	0.51^***^	0.11^**^	0.09	−0.03	0.10	0.14^***^	0.06	–						
13. Peer-reported bullying	0.03	−0.03	0.34^***^	−0.16^***^	0.11^**^	0.47^***^	0.38^***^	0.07	0.35^***^	0.49^***^	0.12^**^	0.12^**^	–					
14. Proactive aggression	0.05	0.05	0.25^***^	−0.07	0.14^***^	0.41^***^	0.53^***^	0.13^**^	0.27^***^	0.55^***^	0.22^***^	0.14^***^	0.64^***^	–				
15. Reactive aggression	0.25^***^	0.41^***^	−0.09	0.30^***^	0.04	0.05	0.14^***^	0.41^***^	0.04	0.10	−0.03	0.01	0.14^***^	0.21^***^	–			
16. Indirect aggression	0.05	−0.13^***^	0.46^***^	−0.27^***^	0.09	0.42^***^	0.26^***^	0.03	0.51^***^	0.31^***^	0.11^**^	0.15^***^	0.59^***^	0.41^***^	0.05	–		
17. Direct aggression	0.06	−0.02	0.35^***^	−0.21^***^	0.19^***^	0.49^***^	0.50^***^	0.12^**^	0.32^***^	0.64^***^	0.20^***^	0.18^***^	0.66^***^	0.77^***^	0.13^***^	0.49^***^	–	
18. Alcohol use	0.02	−0.10	0.27^***^	−0.16^***^	0.15^***^	0.16^***^	0.16^***^	−0.05	0.19^***^	0.18^***^	0.51^***^	0.08	0.18^***^	0.21^***^	−0.04	0.15^***^	0.20^***^	–
*M* (*SD*)	1.40 (0.47)	0.00 (0.99)	0.00 (0.99)	−0.00 (0.99)	1.28 (0.34)	−0.00 (0.99)	−0.01 (0.98)	−0.00 (0.98)	−0.01 (0.90)	−0.00 (0.93)	0.15 (0.46)	1.24 (0.37)	0.01 (0.98)	0.01 (1.00)	0.02 (0.98)	0.01 (0.89)	−0.01 (0.91)	0.28 (0.65)

Ns range 615 to 804. Alcohol use measured via self-report. All other variables measured via peer nominations unless otherwise stated. Variables 1–4 used in the LPA to identity victim subgroups. Variables 1–11 Time 1. Variables 12–18 Time 2

** *p* < 0.01.

*** *p* < 0.001

**Table 3:** Comparison of dependent variables by victim subgroups

		Victims		Non-victims	
		High-status victims (*n* = 63)	Lower-status victims (*n* = 282)	High-status non-victims (*n* = 78)	Lower-status non-victims (*n* = 381)
Bullying					
Self-report	T1	1.57 (0.04)^a^	1.36 (0.02)^b^	1.24 (0.04)^c^	1.17 (0.02)^c^
	T2	1.44 (0.05)^a^	1.40 (0.05)^a,b^	1.20 (0.04)^b,c^	1.17 (0.02)^c^
Peer-report	T1	1.03 (0.11)^a^	−0.18 (0.05)^b^	0.70 (0.10)^a^	−0.18 (0.05)^b^
	T2	0.73 (0.13)^a^	−0.18 (0.06)^b^	0.54 (0.12)^a^	−0.08 (0.05)^b^
Proactive aggression	T1	0.85 (0.11)^a^	−0.05 (0.05)^c^	0.38 (0.10)^b^	−0.19 (0.05)^d^
	T2	0.56 (0.13)^a^	−0.10 (0.06)^b^	0.40 (0.12)^a^	−0.08 (0.05)^b^
Reactive aggression	T1	0.06 (0.12)^a,b^	0.37 (0.06)^a^	−0.26 (0.11)^b^	−0.24 (0.05)^b^
	T2	−0.06 (0.13)^b^	0.35 (0.06)^a^	−0.25 (0.12)^b^	−0.15 (0.05)^b^
Indirect agression	T1	1.00 (0.10)^a^	−0.21 (0.05)^c^	0.64 (0.09)^b^	−0.15 (0.04)^c^
	T2	0.87 (0.11)^a^	−0.22 (0.05)^b^	0.69 (0.10)^a^	−0.11 (0.04)^b^
Direct aggression	T1	0.93 (0.10)^a^	−0.14 (0.05)^b^	0.71 (0.09)^a^	−0.19 (0.04)^b^
	T2	0.72 (0.11)^a^	−0.17 (0.05)^b^	0.38 (0.10)^a^	−0.07 (0.04)^b^
Alcohol use	T1	0.34 (0.06)^a^	0.11 (0.04)^b^	0.41 (0.06)^a^	0.11 (0.04)^b^
	T2	0.72 (0.10)^a^	0.20 (0.06)^b^	0.56 (0.09)^a^	0.24 (0.06)^b^

Predicted adjusted means and standard errors from the multilevel mixed-effects linear regression analyses predicting the dependent variables are reported. All regression models controlled for gender. T1 = time 1. T2 = time 2. Proactive, reactive, indirect and direct aggression are measured via peer nominations. Alcohol use is measured via self-reports. Means in the same row that do not share superscripts (^a, b, c,^ or ^d^) differ at *p* < 0.05 using Holm–Bonferroni adjusted *p*-values for multiple comparisons.

## Data Availability

The data for this manuscript will be made publicly available upon acceptance.

## Footnotes

1 Three lower-status victim groups from the previous study (convergent, self-identified, and peer-identified; Malamut et al., 2021) were combined to create this group. To verify whether this was appropriate, the three groups were compared on all outcome variables (see Table 1 in Supplemental Materials). All three lower-status victim groups followed the same pattern (low or high) for each outcome, with the exception of reactive aggression (convergent victims scored particularly high) and self-reported bullying (peer-identified victims scored low).
