# Supplementary material for: Differences in Aggression and Alcohol Use among Youth with Varying Levels of Victimization and Popularity Status
Source: J Youth Adolesc. Author manuscript; Available in PMC 2022 Oct 1. (PMC9363477; doi:10.1007/s10964-022-01649-7)
Supplement: Table S1 [file NIHMS1821828-supplement-Table_S1.docx]

**Supplemental Materials**

**Differences in Aggression and Alcohol Use among Youth with Varying Levels of Victimization and Popularity Status**

**Table S1.** Comparison of Dependent Variables between Three Subgroups of Lower-Status Victims

Table S1
*Comparison of Dependent Variables between Three Subgroups of Lower-Status Victims*

|  |  |  | Lower-Status Victims | | |
| --- | --- | --- | --- | --- | --- |
|  |  |  | Convergent  (*n* = 31) | Self-identified  (*n* = 186) | Peer-identified  (*n* = 65) |
| Bullying |  |  |  |  |  |
| Self-report | T1 |  | 1.33 (0.34) ^a^ | 1.46 (0.40) ^a^ | 1.09 (0.16) ^b^ |
|  | T2 |  | 1.20 (0.31) ^a,b^ | 1.38 (0.50) ^a^ | 1.12 (0.30) ^b^ |
| Peer-report | T1 |  | -0.17 (1.02) ^a,b^ | -0.11 (0.81) ^a^ | -0.42 (0.36) ^b^ |
|  | T2 |  | 0.01 (0.97) ^a^ | -0.16 (0.70) ^a^ | -0.33 (0.40) ^a^ |
| Proactive aggression | T1 |  | 0.00 (1.07) ^a^ | -0.04 (0.96) ^a^ | -0.15 (0.79) ^a^ |
|  | T2 |  | 0.15 (1.23) ^a^ | -0.11 (0.75) ^a^ | -0.21 (0.65) ^a^ |
| Reactive aggression | T1 |  | 2.29 (1.85) ^a^ | 0.05 (0.86) ^b^ | 0.37 (1.31) ^b^ |
|  | T2 |  | 1.41 (1.80) ^a^ | 0.16 (1.08) ^b^ | 0.42 (1.37) ^b^ |
| Indirect agression | T1 |  | -0.16 (0.61) ^a^ | -0.10 (0.82) ^a^ | -0.53 (0.39) ^b^ |
|  | T2 |  | -0.15 (0.67) ^a^ | -0.11 (0.76) ^b^ | -0.51 (0.24) ^b^ |
| Direct aggression | T1 |  | -0.12 (1.05) ^a^ | -0.10 (0.83) ^a^ | -0.36 (0.74) ^a^ |
|  | T2 |  | -0.09 (1.14) ^a^ | -0.11 (0.74) ^a^ | -0.41 (0.63) ^a^ |
| Alcohol Use | T1 |  | 0.03 (0.18) ^a,b^ | 0.14 (0.37) ^a^ | 0.02 (0.12) ^b^ |
|  | T2 |  | 0.09 (0.29) ^a,b^ | 0.24 (0.56) ^a^ | 0.02 (0.14) ^b^ |

*Note.* Predicted adjusted means and standard errors from the multilevel mixed-effects linear regression analyses predicting the dependent variables are reported. All regression models controlled for gender. T1 = time 1. T2 = time 2. Proactive, reactive, indirect and direct aggression are measured via peer nominations. Alcohol use is measured via self-reports. Means in the same row that do not share superscripts (^a^ or ^b^) differ at *p* < .05 using Holm-Bonferroni adjusted *p*-values for multiple comparisons.
